# A Causal Role for the Cortical Frontal Eye Fields in Microsaccade Deployment

**DOI:** 10.1371/journal.pbio.1002531

**Published:** 2016-08-10

**Authors:** Tyler R. Peel, Ziad M. Hafed, Suryadeep Dash, Stephen G. Lomber, Brian D. Corneil

**Affiliations:** 1 The Brain and Mind Institute, University of Western Ontario, London, Ontario, Canada; 2 Graduate Program in Neuroscience, University of Western Ontario, London, Ontario, Canada; 3 Physiology of Active Vision Laboratory, Werner Reichardt Centre for Integrative Neuroscience, Tuebingen University, Tuebingen, Germany; 4 Department of Physiology & Pharmacology, University of Western Ontario, London, Ontario, Canada; 5 Department of Psychology, University of Western Ontario, London, Ontario, Canada; 6 Robarts Research Institute, London, Ontario, Canada; Yeshiva University Albert Einstein College of Medicine, UNITED STATES

## Abstract

Microsaccades aid vision by helping to strategically sample visual scenes. Despite the importance of these small eye movements, no cortical area has ever been implicated in their generation. Here, we used unilateral and bilateral reversible inactivation of the frontal eye fields (FEF) to identify a cortical drive for microsaccades. Unexpectedly, FEF inactivation altered microsaccade metrics and kinematics. Such inactivation also impaired microsaccade deployment following peripheral cue onset, regardless of cue side or inactivation configuration. Our results demonstrate that the FEF provides critical top-down drive for microsaccade generation, particularly during the recovery of microsaccades after disruption by sensory transients. Our results constitute the first direct evidence, to our knowledge, for the contribution of any cortical area to microsaccade generation, and they provide a possible substrate for how cognitive processes can influence the strategic deployment of microsaccades.

## Abstract 2

Our eyes are constantly in motion. This study shows that the frontal eye fields (an area of the frontal cortex) contribute to the strategic deployment of microsaccades, which aid vision by precisely translating retinal images by a few photoreceptors.

## Author Summary

Microsaccades are small, fixational eye movements that precisely relocate the visual axis. Despite evidence that microsaccades can be strategically controlled in high-acuity visual tasks, impacting visual processing, and considerable knowledge about how microsaccades are generated by the oculomotor brainstem, little is known about the cortical substrates that control microsaccades. To address this gap, we examined microsaccades generated by non-human primates before, during, and after large-volume reversible unilateral or bilateral inactivation of the frontal eye fields, a key oculomotor area in the frontal cortex. In support of a role for the frontal eye fields in microsaccades, microsaccade metrics and kinematics were altered during frontal eye fields inactivation. More surprisingly, frontal eye fields inactivation also impaired the generation of microsaccades following presentation of peripheral cues, regardless of the side of the cue or inactivation configuration. To our knowledge, our results constitute the first direct evidence for the contribution of any cortical area to microsaccade generation and suggest that the frontal eye fields can provide the top-down signals to the oculomotor brainstem needed to strategically guide microsaccades.

## Introduction

Microsaccades, which frequently occur during gaze fixation, translate retinal images by only a few photoreceptors. Despite their modest size, microsaccades strongly impact visual perception [1–5] and visually guided behavior [6–8]. Indeed, visual responses in a number of brain structures are dynamically influenced by either the production or consequence of microsaccades, with responses being enhanced immediately before microsaccades [9], suppressed during or just after microsaccades [7,10,11], and then subsequently enhanced [10–15]. While some mechanisms underlying microsaccade generation have been elucidated in the superior colliculus (SC) [16–20], cerebellum [21], and brainstem saccadic burst generator [22–24], no study has addressed the involvement of any cortical area in microsaccade generation. This gap in knowledge is all the more surprising given the strategic deployment of microsaccades in tasks requiring high visual acuity [25,26], the impacts of microsaccades on visuomotor processing noted above, and the interest in microsaccades as a potential biomarker for visuospatial attention [27–31].

Here, we directly examined the causal role of the frontal eye fields (FEF), a key cortical oculomotor structure that projects strongly to the SC [32,33], in microsaccade generation. To address this, we reversibly inactivated large volumes of either the unilateral or bilateral FEF using cryoloops implanted in the arcuate sulcus and examined the changes in microsaccade behavior, focusing primarily on how FEF inactivation alters the well-known evolution of microsaccades that occurs following peripheral stimulus onset [28,29,34,35]. Our results show that the role for the FEF in microsaccades is distinct from that for the SC, and that the FEF provides a plausible substrate for how microsaccades can be strategically deployed.

## Results

The FEF was reversibly inactivated using cryogenic techniques either unilaterally (three monkeys) or bilaterally (two of the three monkeys) while monkeys performed delayed visually or memory-guided saccades (see Materials and Methods). These tasks required the monkeys to maintain fixation before and after peripheral cues were presented, allowing us to study pre- and post-cue microsaccadic modulations during otherwise steady fixation. We analyzed 74,650 microsaccades from 44,225 trials across monkeys, sessions (i.e., pre-, peri-, and post-cooling), and inactivation configurations (i.e., left or right unilateral inactivation and bilateral inactivation). In this paper, we present representative results from monkey DZ during unilateral inactivation of the left FEF (7,791 trials) or bilateral FEF inactivation (7,378 trials), and we also summarize results from all monkeys. To ensure that the effects of FEF inactivation were not due to satiation or other time-dependent factors, we combined pre- and post-cooling trials into the “FEF warm” condition and compared it to the “FEF cool” condition.

As expected, large-volume FEF inactivation impacted many aspects of (large) saccadic behavior. In a previous report [36], we described the effects of unilateral cryogenic FEF inactivation on immediate and delayed saccades to peripheral targets located 4° or more from the fixation point. Briefly, unilateral FEF inactivation increased reaction times for delayed visually or memory-guided saccades in either direction and decreased accuracy and peak velocity (i.e., decreased the velocity-amplitude main sequence relationship) of contralesional but not ipsilesional saccades. These effects were replicated in the current study and are consistent with the geometry and positioning of the cryogenic loops within the arcuate sulcus relative to FEF’s topography [36,37]. We also found that bilateral FEF inactivation exacerbated all effects, such that saccades in either direction were generated at increased reaction times with lower peak velocities and accuracy. Despite these effects, the monkeys continued to perform well, with error rates increasing by less than 10%. Having established this, we now turn to the specific effects of FEF inactivation on microsaccades.

### FEF Inactivation Increased Microsaccade Amplitude and Decreased Microsaccade Peak Velocity

**Fig 1:**
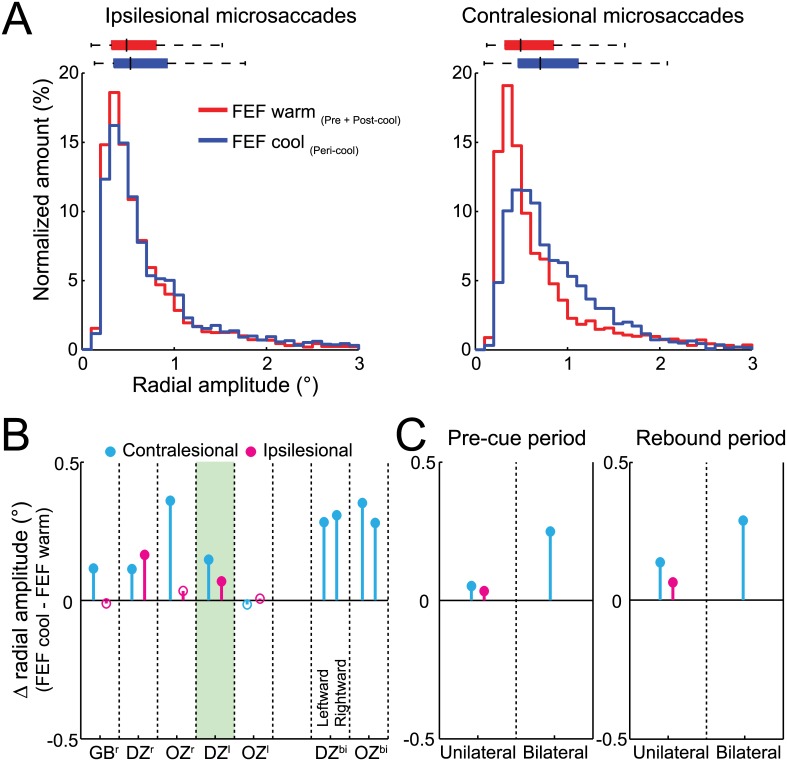
FEF inactivation increased ipsilesional and contralesional microsaccade amplitudes independently of peripheral cueing. (**A**) Unilateral (left) FEF inactivation shifted distributions toward larger amplitudes for each microsaccade direction from example monkey DZ, although we observed larger increases for contralesional microsaccades. Colored bars above the distributions indicate the median, with 25th and 75th percentiles with whiskers extending outward to the 1st and 99th percentiles. (**B**) Microsaccade amplitudes increased for each monkey (GB, DZ, and OZ) and unilateral (X^r^ or X^l^) or bilateral (X^bi^) inactivation configurations, but unilateral FEF inactivation more consistently increased contralesional amplitudes. The shaded area in **B** indicates microsaccades from our example monkey. (**C**) Across monkeys, unilateral or bilateral FEF inactivation increased microsaccade amplitudes in both the pre-cue (left) and rebound (right) periods. Filled symbols in **B** and **C** indicate statistically significant differences using a Wilcoxon rank sum test (*p* < 0.05). Data in Supporting Information (see S1 Data).

Across our sample, we found consistent alterations in microsaccade amplitude and peak velocity, regardless of whether the microsaccade was generated before or after peripheral cue onset in our tasks. Fig 1A shows the effects of unilateral FEF inactivation on ipsilesional and contralesional microsaccade amplitude for our representative dataset. When the FEF was not inactivated, microsaccades had relatively small amplitudes (median: 0.51°) compared to the fixation window, which likely relates to the small size of our fixation cue [38]. During FEF inactivation, the amplitude distributions for both ipsi- and contralesional microsaccades were shifted toward larger amplitudes (*p* < 0.0001, Wilcoxon rank sum test), with greater shifts for contralesional (increased by 21%) versus ipsilesional (increased by 10%) microsaccades. Regardless of increases in microsaccade amplitude during FEF inactivation, the vast majority of microsaccades remained <1.5°. Across our sample, contralesional microsaccade amplitudes increased significantly in four of five configurations; ipsilesional microsaccade amplitude increased significantly in two of five configurations (Fig 1B). During bilateral FEF inactivation, the amplitudes of both leftward and rightward microsaccades also increased significantly (Fig 1B). Importantly, microsaccade amplitude increased regardless of whether microsaccades were generated before or after cue presentation (Fig 1C, and see Materials and Methods for definitions of pre-cue and rebound periods).

Could these increases in microsaccade amplitude be a simple consequence of a biased fixation position? We analyzed fixation position with and without FEF inactivation and found that unilateral FEF inactivation biased fixation position by less than 1° toward the intact visual hemifield (S1A Fig). However, this bias persisted before, during, and after cue presentation (S1B and S1C Fig), meaning that any changes in microsaccade behavior due to FEF inactivation were not sufficient to correct for a biased fixation position. This observation is consistent with the idea that FEF inactivation introduces a new balance point for eye position, as observed during SC inactivations [19,39], rather than a mechanism that acts to correct for the biased fixation position, since unilateral FEF inactivation also increased ipsilesional microsaccade amplitudes (Fig 1). Similarly, we observed increased microsaccade amplitudes in both directions during bilateral FEF inactivation (Fig 1B and 1C), despite a fixation position bias only toward one side (S1C Fig).

**Fig 2:**
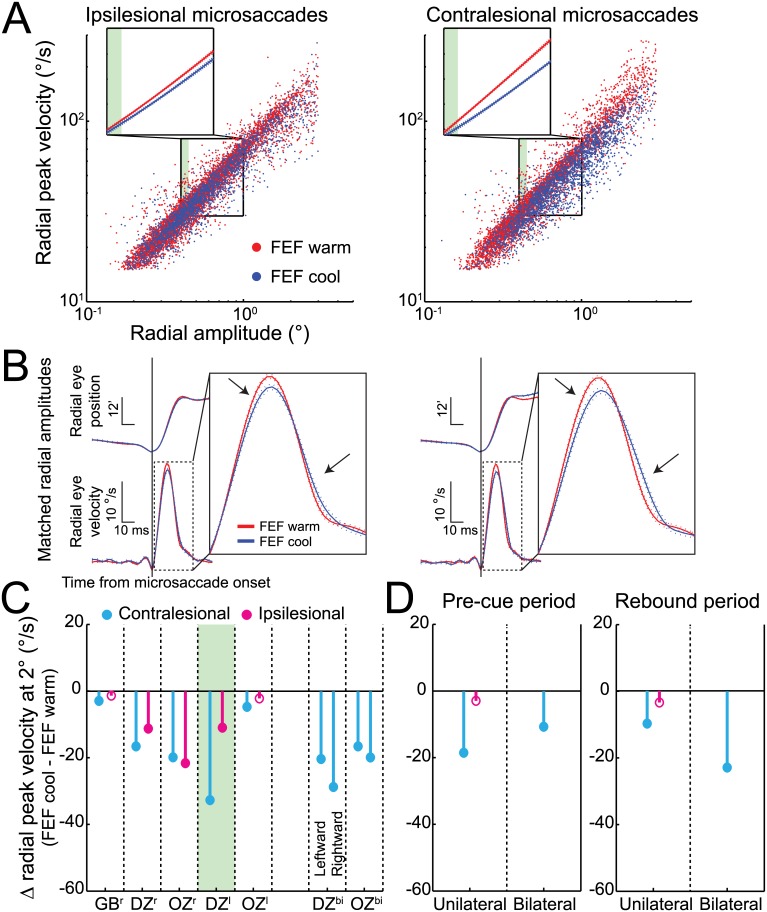
FEF inactivation decreased ipsilesional and contralesional microsaccade peak velocities, both before and after cue onset. (**A**) Unilateral (left) FEF inactivation reduced peak velocity for contralesional microsaccades independently of amplitude in our example monkey DZ and also decreased peak velocities for ipsilesional microsaccades. As shown in each inset, decreased peak velocities were associated with a downward shift in the main sequence relationship (+/- 95% confidence intervals). (**B**) FEF inactivation reduced peak velocity for microsaccades matched for radial amplitude, here shown for monkey DZ by averaging radial eye position and velocity traces (+/- standard error) aligned to microsaccade onset. As indicated by the shaded regions in **A**, we selected ipsilesional and contralesional microsaccades having radial amplitudes between 0.40° and 0.45°. The bilateral influence of FEF inactivation on amplitude-matched microsaccades is demonstrated by decreased peak velocity and increasing duration within the enlarged radial velocity traces (see arrows). (**C**) Peak velocity extracted at 2° decreased for contralesional microsaccades across monkeys and inactivation configurations and occasionally decreased for ipsilesional microsaccades. Distributions of peak velocities at 2° were obtained by bootstrapping 5,000 random samples of microsaccades and extracting the peak velocity at 2° from each linear regression. (**D**) Across monkeys, unilateral and bilateral FEF inactivation produced similar decreases in contralesional peak velocity at 2° in both the pre-cue and rebound periods. Filled symbols in **C** and **D** indicate statistically significant differences using a Welch's *t* test (*p* < 0.05) with 5,000 bootstrapped samples from each of the FEF warm and FEF cool conditions. Data in Supporting Information (see S2 Data).

More compelling evidence against a simple compensatory mechanism based on a bias in fixation position is provided by microsaccade peak velocity, which decreased independent of increased microsaccade amplitude or fixation offset. Such decreases in peak velocity are shown in the velocity-amplitude main sequence relationships in Fig 2A for both ipsilesional and contralesional microsaccades; note how both main sequence relationships are shifted downward during unilateral FEF inactivation. To determine the significance of such changes, we fitted a linear regression to 5,000 bootstrapped samples of microsaccades for both the FEF warm and FEF cool conditions and then extracted peak velocities from each relationship at amplitudes of 0.4° to 1.0° with 0.1° increments. We found significantly decreased peak velocities across this entire range of amplitudes for both ipsilesional and contralesional microsaccades (insets of Fig 2A, each *p* < 0.01, Welch's *t* tests). Fig 2B shows how FEF inactivation alters the kinematic profiles of microsaccades matched for radial amplitudes (e.g., between 0.40° and 0.45°; see shaded region of Fig 2A) by lowering peak velocity and significantly increasing microsaccade duration (ipsilesional, *p* < 0.05; contralesional, *p* < 0.0001, Wilcoxon rank sum test). Such changes in kinematics and duration are consistent with FEF inactivation altering the drive to brainstem circuits generating microsaccades. To analyze any changes across our sample, we extracted peak velocities at 2° and found significant decreases of 9% and 23% for ipsilesional and contralesional microsaccades during FEF inactivation, respectively (Fig 2C, each *p* < 0.0001, Welch's *t* tests). Unilateral FEF inactivation significantly decreased contralesional peak velocity in all five configurations and significantly decreased ipsilesional peak velocity in three of five configurations (Fig 2C). Bilateral FEF inactivation significantly decreased peak velocity for both leftward and rightward microsaccades in both monkeys (Fig 2C). Once again, such bilateral decreases in microsaccade peak velocity occurred regardless of whether microsaccades were generated in the pre-cue or rebound period (Fig 2D) and despite a unilateral bias in fixation position during bilateral inactivation (S1C Fig).

Therefore, even prior to any task-related stimulus, FEF inactivation had a measurable impact on microsaccade metrics and kinematics, with such an impact often influencing even ipsilesional microsaccades. We next describe an even larger impact of FEF inactivation on the rate of cue-induced microsaccades.

### FEF Inactivation Blunted the Rate of Cue-Induced Microsaccades

**Fig 3:**
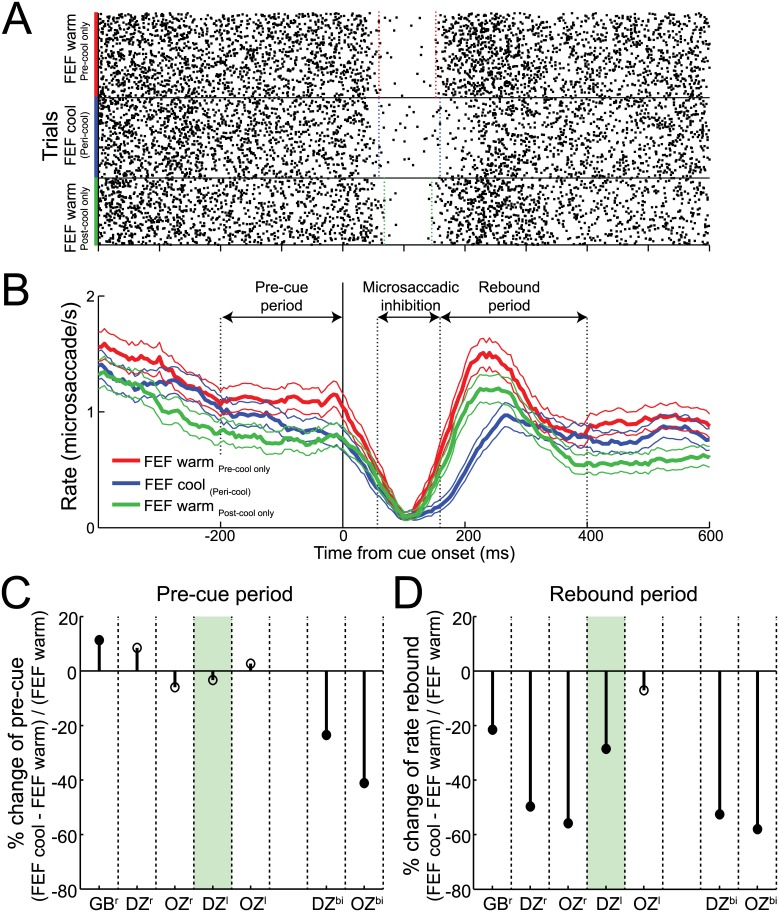
FEF inactivation markedly reduced microsaccades in the rebound period. (**A**) Microsaccade onset times relative to cue onset for individual pre-, peri-, and post-cooling trials from our example monkey with a unilateral (left) FEF inactivation. Each dot is a microsaccade onset time, and each row is a trial. (**B**) Corresponding time-courses of mean microsaccade rate (+/- 95% confidence intervals) for each of the pre-, peri-, and post-cooling sessions. In our example monkey, unilateral FEF inactivation exerted its greatest impact on microsaccade rate during the rebound period (i.e., 140–400 ms after cue onset), with no changes occurring in the pre-cue period (i.e., 200 ms period before cue onset) or the microsaccadic inhibition period (i.e., 60–140 ms after cue onset). (**C**) Across monkeys, we found consistent microsaccade rate decreases in the pre-cue period with bilateral but not unilateral inactivation configurations. (**D**) In contrast, both unilateral and bilateral FEF inactivation consistently decreased microsaccade rate in the rebound period. Same format as Fig 2C. Data in Supporting Information (see S3 Data).

Microsaccade rate shows robust and highly repeatable modulations after peripheral cue onsets, decreasing ~50 ms after cue onset and then rebounding before returning to baseline [4,29,35]. To analyze the effects of FEF inactivation on microsaccade rate, we divided our data into three periods: pre-cue, microsaccadic inhibition, and rebound (Fig 3; see Materials and Methods for how these periods were defined; microsaccade rate was calculated within a sliding ±50 ms window with step size of 5 ms). As shown in our representative data, the influence of unilateral FEF inactivation was largely specific to the rebound period (Fig 3A and 3B): note how the rate of such rebound microsaccades decreased from 1.08 to 0.70 microsaccades/s with FEF inactivation and then recovered to 0.87 microsaccades/s with FEF rewarming (both changes significant, *p* < 0.0001, Welch's *t* test). In contrast, microsaccade rate in the pre-cue period decreased from 1.11 to 0.91 microsaccades/s (*p* < 0.0001) with FEF inactivation and decreased further to 0.76 microsaccades/s (*p* < 0.01) when the FEF was rewarmed, suggesting that this effect may have been due to satiation with increasing trial count. Microsaccade rate during the inhibition period was unchanged. We also analyzed both the start and end of microsaccadic inhibition and the timing of the first rebound microsaccade after cue presentation (see Materials and Methods). FEF inactivation had no influence on the start of microsaccadic inhibition following cue onset (59 ms for both pre- and peri-cool) or its end (152 versus 158 ms for FEF pre- versus peri-cool, *p* = 0.74, Wilcoxon rank sum test). In contrast, the timing of the first rebound microsaccades increased from 264 to 291 ms with FEF inactivation and then recovered to 266 ms when the FEF was rewarmed (S2A Fig, both *p* < 0.0001; Wilcoxon rank sum test).

For each of the three monkeys, unilateral FEF inactivation systematically decreased microsaccade rate during the rebound period (Fig 3D, significant in four of five configurations) but not the pre-cue period (Fig 3C). Bilateral FEF inactivation further reduced microsaccade rate during the rebound period (Fig 3D) but, unlike unilateral inactivation, also significantly decreased microsaccade rate during the pre-cue period (Fig 3C). Thus, with bilateral inactivation, there was a generalized decrease in microsaccades. Across our sample, the decrease in microsaccade rate in the rebound period averaged 24% with unilateral inactivation and 54% with bilateral inactivation. Consistent with a generally exacerbated effect of bilateral versus unilateral FEF inactivation, we also found a relatively greater increase in the timing of the first microsaccade in the rebound period during bilateral (44 ms) versus unilateral (7 ms; S2B Fig). These results indicate that FEF integrity is critical for cue-induced microsaccades and that larger bilateral inactivation volumes can further impact microsaccades generated before cue presentation. These effects on cue-induced microsaccade rate are also categorically different from those reported during pharmacological inactivation of the SC, where cue-induced microsaccade rates remained unchanged [18].

**Fig 4:**
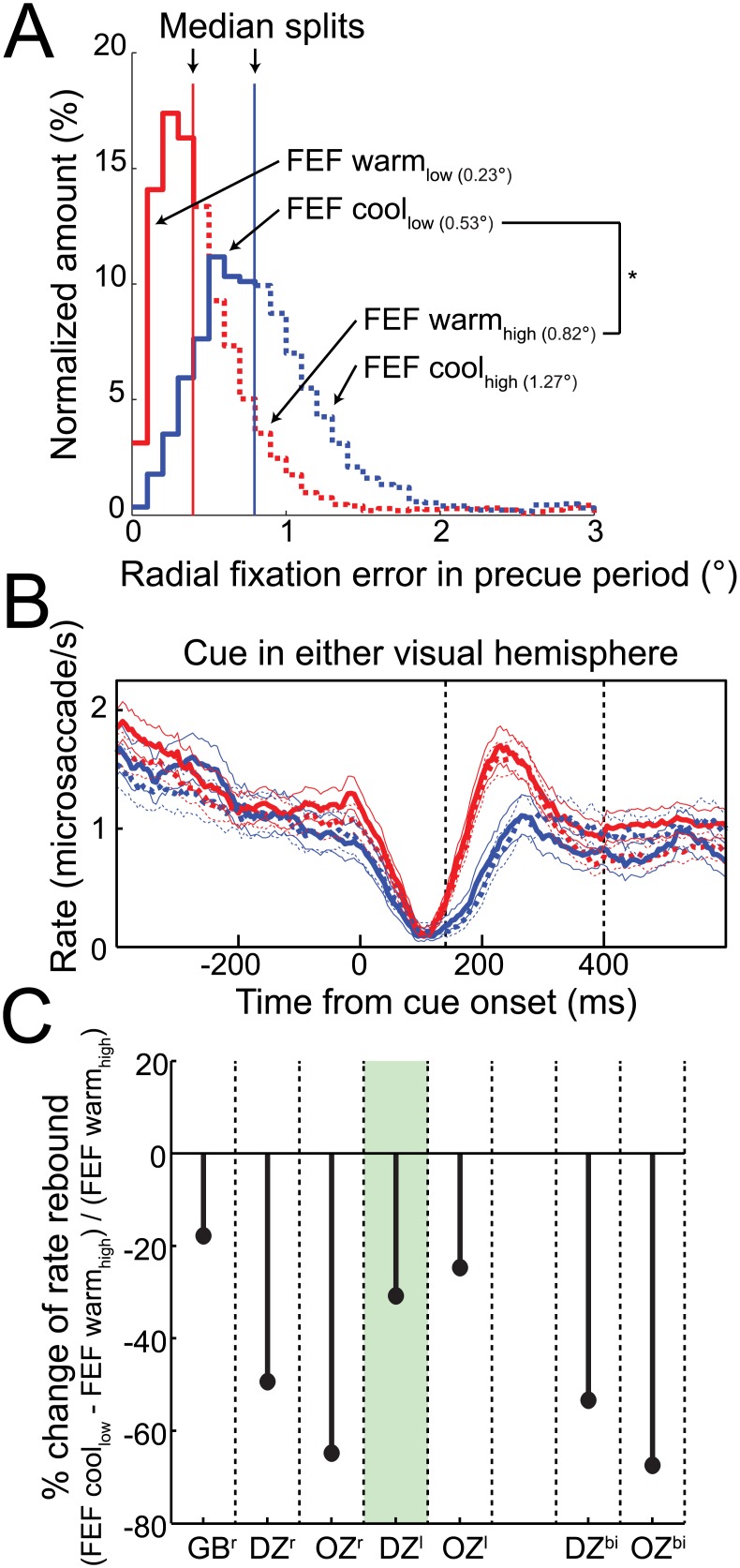
FEF inactivation decreased rebound microsaccades despite increases in fixation error. (**A**) Unilateral (left) FEF inactivation increased radial fixation error before cue onset in our example monkey DZ, largely due to shift in fixation position toward the intact side (see S1 Fig). (**B**) For this same monkey, FEF inactivation reduced microsaccade rate in the rebound period for both trials with high (higher-than- median values) and low (lower-than-median values) radial fixation error in the pre-cue period. Importantly, FEF inactivation reduced rebound microsaccades despite a significantly greater fixation error in FEF warm_high_ (0.82°) compared to the FEF cool_low_ (0.53°). (**C**) Across all monkeys and cooling configurations, FEF inactivation consistently reduced microsaccade rate in the rebound period when comparing FEF warm_high_ and FEF cool_low_ trials. Same format as Fig 2C. Data in Supporting Information (see S4 Data).

Because FEF inactivation also introduced a bias in fixation position, we wondered whether this could explain the changes in microsaccade rate during the rebound period. To investigate this, we repeated our analysis of microsaccade rate after performing a median split of FEF warm and FEF cool trials based on their radial fixation error in the pre-cue period (Fig 4A). This analysis exploits the substantial overlap in fixation positions with or without FEF inactivation (S1A Fig), and, in fact, fixation error was significantly larger for the higher-than-median FEF warm trials than for the lower-than-median FEF cool trials (Fig 4A, fixation error for FEF warm_high_ = 0.82°; fixation error for FEF cool_low_ = 0.53°). As shown in Fig 4B, the robust decrease in rebound microsaccades during FEF inactivation persisted regardless of this split in fixation error. To quantify this across our sample, we calculated the change in rebound microsaccades from FEF warm_high_ trials to FEF cool_low_ trials. If the changes in rebound microsaccades during inactivation arose because of a greater fixation error, then we should observe no decrease in rebound microsaccades across these subsets of data, because fixation offset was greater in FEF warm_high_ trials. However, as shown in Fig 4C, we still observed a profound decrease in microsaccade rate during the rebound period with FEF inactivation. Therefore, the effects of Fig 3 cannot be due to fixation error.

Finally, these analyses led us to investigate whether FEF inactivation impacted eye position drift, and not just overall bias. Even though our eye tracker was not well suited to study drift at a higher resolution, to the extent that we could measure it, eye position drift before cue onset was not influenced by FEF inactivation (S3 Fig). FEF inactivation also did not influence dependencies between drift and microsaccades. Specifically, we analyzed the relationship between eye position drift and microsaccades, as previous work has shown that drift speed is lower before as compared to after a microsaccade [40]. However, we observed no systematic influence of FEF inactivation on this relationship (S4 Fig). Thus, we conclude that the effects shown in Fig 3 on FEF inactivation on microsaccade rate could not be attributed to biases in fixation position or drift in the pre-cue period.

### Unilateral FEF Inactivation Decreased Microsaccade Rate Regardless of the Side of the Cue

**Fig 5:**
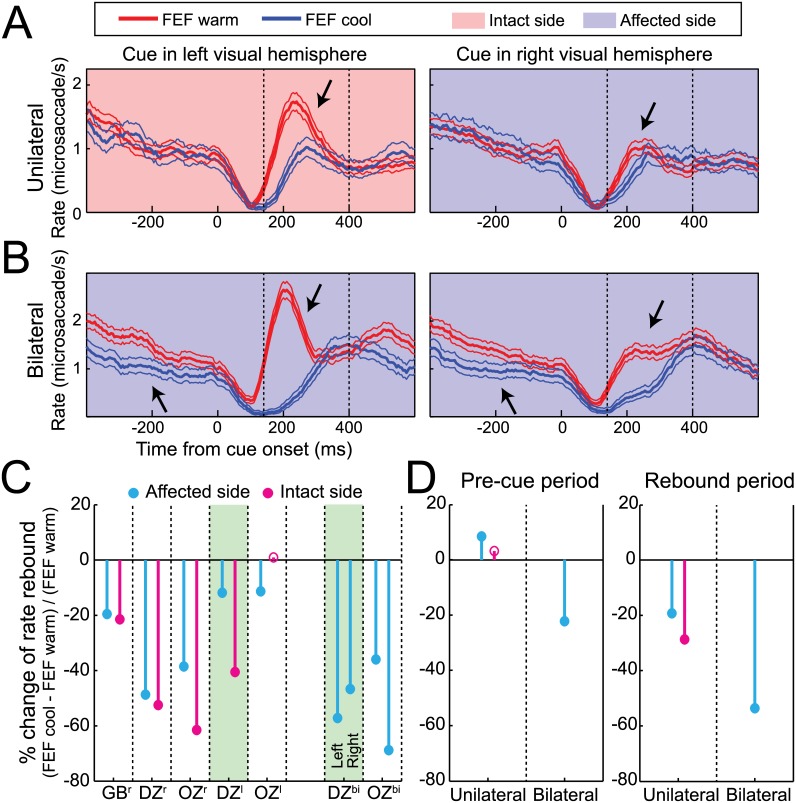
FEF inactivation decreased microsaccade rate in response to cues appearing in either visual hemifield. (**A**) Time courses of mean microsaccade rate (+/- 95% confidence intervals) in response to cues either in the affected or intact visual hemifield for unilateral FEF warm and FEF cool conditions from our example monkey DZ. As indicated by arrows, unilateral (left) FEF inactivation decreased microsaccade rate in the rebound period for cues appearing in both the intact and affected visual hemifield. (**B**) Bilateral FEF inactivation produced similar, but quantitatively larger, decreases in microsaccade rate in response to cues in either affected visual hemifield from this same monkey (downward arrows). Bilateral FEF inactivation also decreased microsaccade rate in the pre-cue independent of subsequent cue location (upward arrows). (**C**) Microsaccade rate in the rebound period consistently decreased for both the intact and affected side across monkeys and inactivation configurations, with somewhat larger effects accompanying bilateral versus unilateral FEF inactivation. (**D**) Across monkeys, only bilateral FEF inactivation decreased microsaccade rate in the pre-cue period and had a quantitatively larger impact on rate in the rebound period compared to unilateral FEF inactivation. Same format as Fig 2C. Data in Supporting Information (see S5 Data).

Next, we investigated whether the effects of FEF inactivation on microsaccade rate only occurred when cues were presented contralateral to the side of inactivation. To our surprise, we found that unilateral FEF inactivation decreased microsaccade rate during the rebound period regardless of the side of the cue (Fig 5A, shown for our representative data during left FEF inactivation). Despite an idiosyncratically higher rate of rebound microsaccades for cues presented in the intact (left) hemifield even before inactivation, unilateral FEF inactivation significantly reduced microsaccade rate during the rebound period for cues presented in both the intact (left; 41% decrease, *p* < 0.001, Welch’s *t* test) and affected (right; 12% decrease, *p* < 0.05) hemifield (see arrows). We observed such robust decreases in microsaccade rate during the rebound period during unilateral FEF inactivation across our sample, regardless of the side of the cue relative to the side of inactivation (Fig 5C). Such decreases were even greater during bilateral FEF inactivation (Fig 5B; both hemifields are presumably affected in this configuration; Fig 5C).

We also compared the influence of unilateral or bilateral FEF inactivation on microsaccade rate during the pre-cue and rebound periods (Fig 5D). This analysis again revealed that each cooling configuration robustly decreased the rate of microsaccades in the rebound period regardless of the side of the cue, but that only bilateral FEF inactivation decreased microsaccade rate before cue onset. Together with Fig 3, these results demonstrate the importance of FEF integrity when microsaccades are deployed, particularly after cue onset.

### FEF Inactivation Altered the Directions of Microsaccades

Cue presentation is known to briefly bias microsaccade direction toward and then away from the cue [28,29,34,35]; will FEF inactivation alter such directional modulations? Our monkeys exhibited strong idiosyncratic tendencies in microsaccade direction even before FEF inactivation, which complicates our interpretation. However, we still observed consistencies across our sample, especially when examining how FEF inactivation impacted the fraction of microsaccades toward the cue during the rebound period.

**Fig 6:**
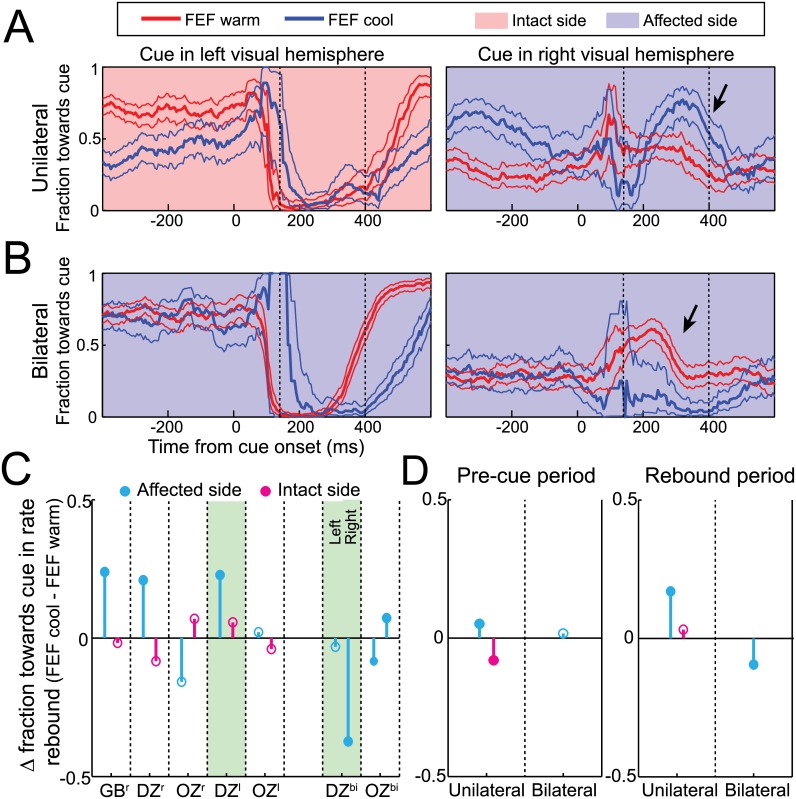
Unilateral FEF inactivation biased microsaccades toward the affected side, whereas bilateral FEF inactivation rescued this effect and delayed and magnified any pre-existing cue-induced directional modulations. (**A**) Time courses of mean microsaccade directionality (i.e., fraction toward cue +/- 95% confidence intervals) in response to cues either in the affected or intact visual hemifield for unilateral FEF warm and FEF cool conditions from our example monkey DZ. While unilateral FEF inactivation biased microsaccades towards the affected field before cue onset, a change in post-cue microsaccades only accompanied cues for the affected visual hemifield (see arrow). (**B**) Bilateral FEF inactivation did not bias microsaccades in the pre-cue period but delayed and magnified the pre-existing directional biases in the microsaccadic inhibition and rebound periods (see arrow). (**C**) Three of five unilateral FEF inactivation configurations had similar effects, whereas bilateral FEF inactivation in both monkeys produced no pre-cue directional bias, and a magnification of pre-existing biases in the rebound period. (**D**) A pre-cue bias toward the affected side was consistent across monkeys only with unilateral FEF inactivation, whereas unilateral and bilateral FEF inactivation impacted cue-induced microsaccade directionality following cues in the affected side. Same format as Fig 2C. Data in Supporting Information (see S6 Data).

In general, unilateral FEF inactivation biased microsaccades toward the affected side, with this bias becoming more pronounced following contralesional cues. For example, before FEF inactivation, monkey DZ had a strong idiosyncratic tendency to make leftward microsaccades, which was perturbed for ~400 ms after cue presentation (Fig 6A). Left FEF inactivation increased the tendency for rightward microsaccades even before cue onset (i.e., the blue line lies above the red line for rightward cues, but below the red line for leftward cues), perhaps to correct for an altered fixation position. During the microsaccade rebound period, FEF inactivation exacerbated this effect only when cues were presented in the affected hemifield (arrow in Fig 6A). To quantify this effect, we measured how FEF inactivation altered the fraction of microsaccades toward cues in the rebound period, segregated by the side of the cue. This fraction significantly increased from 40% to 62% during unilateral FEF inactivation for cues in the affected hemifield (*p* < 0.0001, Welch's *t* test) but only increased from 6% to 11% for cues in the intact hemifield (*p* = 0.08). Across our sample, such an increase was seen in three of five unilateral configurations for cues in the affected hemifield but never for cues presented in the intact hemifield (Fig 6C). Thus, FEF inactivation influenced microsaccade directionality after contralesional but not ipsilesional cues. Interestingly, this directional profile differs from how FEF inactivation influenced microsaccade rate for both contralesional and ipsilesional cues (Figs 3 and 5).

For bilateral FEF inactivation, pre-existing biases in microsaccade direction following cue onset were delayed and magnified. For example, in monkey DZ (Fig 6B), bilateral FEF inactivation did not alter the general idiosyncratic tendency of the monkey (e.g., before or long after cue onset), but it instead prolonged and exaggerated the transient modulation of microsaccades after the cue. Quantitatively, the fraction of microsaccades toward the cue during the rebound period changed from 7% to 4% for leftward cues (*p* = 0.08, Welch's *t* test) and from 45% to 7% for rightward cues (*p* < 0.0001, see arrow).

Across our sample, unilateral FEF inactivation biased microsaccade directions toward the affected side even before cue onset (Fig 6D). This is again different from SC inactivation [18], but it could be related to the altered fixation position. However, the bilateral inactivation data shows that altered fixation position does not produce directional biases in the pre-cue period. Interestingly, changes in microsaccade directionality after cue onset only occurred in the affected hemifield during unilateral inactivation (Fig 6C), but even these changes were not consistently seen in our sample. Taken together, all of our results emphasize that the main effect of FEF inactivation on microsaccade deployment is through modulations of rate rather than microsaccade directionality. This profile differs completely from that seen following inactivation of the SC, which robustly and consistently altered microsaccade direction without influencing microsaccade rate [18].

## Discussion

Our study demonstrates a causal role for the FEF in microsaccade generation, particularly following cue onset. A number of our results are novel, given that this is the first study to our knowledge of any cortical area being involved in microsaccade production. First, unilateral FEF inactivation increased microsaccade amplitude and decreased microsaccade peak velocity, particularly for contralesional microsaccades; this suggests a role for the FEF in contributing to brainstem signals during microsaccade generation, independent of peripheral cues. Second, unilateral FEF inactivation severely impaired cue-induced microsaccades for cues appearing in either hemifield; therefore, changes in microsaccade generation with unilateral FEF inactivation are not the result of impaired processing of a contralateral visual stimulus but rather attest to the FEF's role in deploying microsaccades following any peripheral cue. Third, bilateral FEF inactivation exacerbated the effects of unilateral FEF inactivation, consistent with the FEF contributing to microsaccades generated towards either hemifield. In this discussion, we consider the implications of these results in the context of what is known about microsaccade generation and deployment, known properties of FEF activity, and the emerging view that microsaccades are an essential component of optimal sampling of a visual scene [3,41,42].

### Substrates for Top-Down Control of Microsaccades

Microsaccades can be strategically deployed in tasks requiring high visual acuity [25,26], but the substrates responsible for such top-down influences on microsaccades are poorly understood. Previous studies have demonstrated a critical role for the SC in microsaccade generation and deployment [16,18,19]. Frontal inputs into the SC may enable cognitive processes to influence microsaccade behavior. The results of FEF inactivation, which preferentially impacted the rate of microsaccades following peripheral cue onset, are largely consistent with this idea. Furthermore, the FEF has been implicated in covert visuospatial attention [43,44] and sends visual, cognitive, and saccade-related signals directly to the SC [45]. We are not suggesting that the FEF is the sole source of frontal input into the SC for microsaccade control, but it may well serve as an important interface between the SC and other prefrontal areas.

We were also intrigued by the differences in how FEF or SC inactivation impacted microsaccades generated after peripheral cue onset. Specifically, FEF inactivation decreased microsaccade rate without having an equally large impact on direction; in contrast, inactivation of the caudal SC (which represents peripheral cue locations) impacted microsaccade direction without influencing rate [18]. There are a number of potential, and not mutually exclusive, explanations for these comparative results. They may speak to a particularly important role for the FEF in intervals requiring top-down control of microsaccades, in this case providing signals related to when a microsaccade should be generated. Alternatively, our cryogenic inactivation of the FEF inactivated a much larger volume of tissue compared to the more focal pharmacological techniques used to inactivate the SC (see below). Such differences in inactivation volume are likely even more important considering that the strength of topographic organization in the FEF tends to be less than that observed in the SC. FEF neurons tuned for small retinal errors, which are akin to those found in the rostral SC, tend to be diffusely distributed throughout the FEF and not just confined to the most lateral portion [45,46]. Thus, methodological differences between inactivation techniques hinder the functional conclusions about the comparative role of each area in microsaccade behavior. Nevertheless, the impact of FEF inactivation on the microsaccade rate signature can help us better understand the underlying neural mechanisms.

### Implications of Our Findings on the Microsaccade Rate Signature

The microsaccade rate signature describes the well-known and highly replicable inhibition and then rebound of microsaccade rate following presentation of any stimulus [28,29,34,35,47]. Despite the large volume of inactivated FEF tissue, unilateral inactivation delayed and blunted microsaccade production only during the rebound period (Fig 3), without affecting the baseline rate of microsaccades before cue onset or the start of the microsaccadic inhibition period. Perhaps most surprisingly, such effects were observed regardless of the side of cue presentation. Hence, they cannot simply be explained by impaired processing or neglect of a contralesional stimulus. The temporal specificity of FEF inactivation, impairing the rebound but not baseline or inhibition periods, demonstrates that recovery of microsaccades after disruption by sensory transients requires frontal inputs and, hence, is not simply a passive process. Consistent with this, the direction of microsaccades in the rebound period tends to be opposite to those preceding reflexive microsaccades directed toward the cue [31]. Following the same logic, FEF inputs do not seem to be involved in the onset of microsaccade inhibition, as inhibition onset was not influenced by FEF inactivation. Based on our results, it appears that different portions of the microsaccade rate signature are attributable to different neural substrates (e.g., non-frontal inputs to microsaccade inhibition and frontal inputs to the rebound).

Interestingly, a role for frontal inputs in the first microsaccade after inhibition is consistent with recent models regarding microsaccade generation. In a model by Hafed and Ignaschenkova [35] that utilizes the framework of a recurring rise-to-threshold process, the process initiating the first microsaccade after inhibition has a faster rate of rise to threshold. Similarly, in a model by Engbert [48,49] that considers spatiotemporal dynamics of SC activity, the rebound from inhibition is associated with a change in threshold that integrates sensory and attentional inputs. While both of these models are agnostic as to the source of signals that change microsaccade behavior in the rebound period, attributing either a faster rate of rise (in the Hafed and Ignaschenkova model) or attentional signals (in the Engbert model) to frontal sources is broadly consistent with the impact of FEF inactivation on the microsaccade rate signature. Note, however, that our reasoning regarding the impact of unilateral FEF inactivation on the Engbert model hinges on the assumption that a large unilateral inactivation can produce bilateral effects (see below). In support of our contention that frontal sources are involved in rebound microsaccades, simulations of the Hafed and Ignaschenkova model in which we reduced the rate of rise related to the first microsaccade after inhibition produced results very similar to those produced by FEF inactivation (S1 Text and S5 Fig).

While we observed strong influences of FEF inactivation on microsaccade rate, we observed little systematic influence of FEF inactivation on microsaccade direction. This finding may be attributable to the idiosyncrasies of our subjects, but perhaps more fundamentally, we only studied microsaccades during the performance of delayed-saccade tasks. The strongest evidence linking microsaccade direction to the allocation of visuospatial attention has come from tasks in which covert attention needs to be allocated precisely to perform the task [4,28–30,50]. Recent psychophysical results have demonstrated a dissociation between microsaccade rate and direction effects in attention paradigms [30], and experiments inactivating the SC have shown that rate and direction may not necessarily be affected by the same neural mechanism [18]. In light of these findings, it will be of considerable future interest to see the comparative effect of FEF inactivation on microsaccade rate and direction in other paradigms.

### Unilateral FEF Inactivation Produces Bilateral Effects on Microsaccades

The FEF has an important role in the generation of saccades and deployment of visuospatial attention into the contralateral visual hemifield [43,44], and the effects of FEF inactivation on contralesional microsaccades are consistent with the extension of this role for the FEF into the range of the smallest amplitude saccades. How then do we explain the impact of FEF inactivation on the peak velocity of ipsilesional microsaccades, and on microsaccades deployed after the onset of ipsilesional cues?

The response fields for neurons in the rostral SC can cover portions of both contralateral and ipsilateral fields [16]. If homologous FEF neurons tuned for small amplitudes also cover both hemifields, then inactivation of such neurons may contribute to the decreases in ipsilesional microsaccade peak velocity with unilateral FEF inactivation. Consistent with this, the lateral portion of one FEF, which preferentially represents small amplitude saccades, also projects to the contralateral SC [51]. A preferential projection from the lateral but not medial FEF to the contralateral SC may explain why unilateral FEF inactivation did not decrease the peak velocity of larger ipsilesional saccades [36].

Furthermore, recent findings suggest a more nuanced role in how the FEF contributes to spatially guided behavior. For example, while focal FEF inactivation increases or decreases the reaction times of contralesional or ipsilesional saccades respectively [43,52,53], larger volume temporary or permanent lesions of the FEF raise saccade reaction times bilaterally [36,54]. In our previous work [36], we estimated that the volume inactivated via cooling is conservatively at least four times larger than that typically achieved using pharmacological modulations or optogenetics [18,19,55]. In light of this large inactivation volume, we speculated [36] that bilateral reaction time increases may arise from differences in how the FEF commits to a saccadic decision via widespread disinhibition of the intact FEF or to the presence of diffusely distributed FEF neurons with ipsilateral response fields [56] whose contribution is only revealed by large-volume inactivation. Similarly, inactivation of diffusely distributed FEF neurons tuned to small retinal errors [45,46] may delay the generation of microsaccades during the rebound period, regardless of the side of the cue (e.g., S4 Fig), thereby delaying and blunting the rebound period.

### Conclusions

The FEF has been implicated in the deployment of covert visuospatial attention via top-down signals to extrastriate visual cortex [57–59] and was recently shown to contribute to pupil dilation [60]. Our discovery of a role for the FEF in microsaccade deployment raises the interesting possibility that the FEF can also influence visual processing in still more ways, for example, by strategically deploying microsaccades or via pre-microsaccadic modulations that shape visual processing before the arrival of re-afferent visual input [9]. Our findings set the stage for future experiments that distinguish how cognitive processes optimize visual processing via the preparation and generation of microsaccades or by coordinating such microsaccades with other components of the orienting response [27].

## Materials and Methods

### Subjects and Physiological Procedures

Three male monkeys (*Macaca mulatta*, monkeys GB, DZ, and OZ, weighing 11.1, 9.8, and 8.6 kg, respectively) were used in these experiments. Only monkey GB contributed data to our previous manuscript [36]. All training, surgical, and experimental procedures conformed to the policies of the Canadian Council on Animal Care and National Institutes of Health on the care and use of laboratory animals, and were approved by the Animal Use Subcommittee of the University of Western Ontario Council on Animal Care (2007-099-10). We monitored the monkeys' weights daily, and their health was under the close supervision of the university veterinarians.

Each monkey underwent one surgery to enable reversible cryogenic inactivation of one or both FEFs. Monkeys DZ and OZ were implanted with bilateral FEF cryoloops, whereas monkey GB was only implanted with unilateral FEF cryoloops in the right hemisphere. Our surgical procedures of implanting cryoloops in the arcuate sulcus have been previously described [36,61]. Briefly, we performed a small, 2.25 cm^2^ craniotomy at the stereotaxic coordinates of the arcuate sulcus spur and implanted two customized, stainless steel cryoloops (each 5 to 8 mm in length and extending 3 mm into the sulcus) into each arcuate sulcus, which allowed for the cooling of tissue adjacent to the superior and inferior arms of the arcuate sulcus. Cryoloop temperatures of 3°C silence post-synaptic activity in tissue up to 1.5 mm away without influencing the propagation of action potentials in nearby axons [61]. For this manuscript, we only collected data using the cryoloop in the inferior arm of the arcuate sulcus, which provided an estimated volume of inactivation of 90 mm^3^ in the anterior bank of the arcuate sulcus. Cooling only the cryoloop in the inferior arm of the arcuate sulcus produced the expected triad of contralateral saccadic deficits (i.e., decreases in peak velocity, accuracy, and increases in reaction time), which was approximately 70% of the total saccadic deficits observed from cooling both cryoloops [36].

### Data Collection

Head-restrained monkeys were placed in front of a rectilinear grid of 500+ red LEDs covering ±35° of the horizontal and vertical visual field. We conducted experiments in a dark, sound-attenuated room and recorded each monkey's eye position using a single, chair-mounted eye tracker (EyeLink II). The behavioral tasks were controlled by customized real-time LabView programs on a PXI controller (National Instruments) at a rate of 1 kHz.

A single experimental dataset consisted of a pre-, peri-, and post-cooling session, with each session containing the same number of correct trials. The number of trials for a given dataset ranged from 180 to 480 correct trials, depending upon the number of cue locations. Our experimental procedure for cryogenic inactivation of the FEF has been previously described [36]. Briefly, following the completion of the pre-cooling session, chilled methanol was pumped through the lumen of the cryoloops, decreasing the cryoloop temperature. Once the cryoloop temperature was stable at 3°C for at least 3 min, we began the peri-cooling session. Upon finishing the peri-cooling session, we turned off the cooling pumps, which allowed the cryoloop temperature to rapidly return to normal. When the cryoloop temperature had reached 35°C for at least 3 min, we started the post-cooling session. Because we simultaneously recorded neurons in the intermediate layers of the superior colliculus (iSC) with FEF inactivation, it was necessary to minimize the amount of time for transitions (i.e., shorter than 3 min between pre- and peri-cooling and peri- and post-cooling sessions) to ensure continued isolation of an iSC neuron throughout the full dataset. However, cryoloop temperatures rapidly decreased or increased when the cooling pumps were turned on and off, respectively, and we still found similar effects on saccadic behavior with slightly reduced transition durations. The effects of FEF inactivation on neuronal activity within the iSC will be described in a future manuscript.

### Behavioral Tasks

Monkeys performed memory and visually-guided saccades toward peripheral cues after a delayed response period. Following a variable fixation period of 750 to 1000 ms during which monkeys maintained fixation within a +/- 3° window of a central cue, a peripheral cue appeared in either visual hemifield. Monkeys were required to maintain fixation of the central cue and delay their saccadic response until the central cue was extinguished. Note that despite the large fixation window in our experiments, our central cue was 0.63° in diameter, explaining why most microsaccades were significantly smaller than 1° (Fig 1A). Peripheral cues were either extinguished 150 or 250 ms after onset or remained on for the ensuing memory or visually guided saccade, respectively. After a delayed response period of at least 750 ms, monkeys were rewarded with a liquid reward if they generated a saccade toward the location of the remembered or persistent peripheral cue within 1,000 ms of the offset of the central cue. This response window allowed us to differentiate trials with increased saccade reaction times from neglect of the peripheral cue during FEF inactivation, although monkeys had very few saccade reaction times >500 ms. When we were also recording iSC activity, the location of one peripheral cue coincided with the peak of the response field of an isolated iSC neuron; the other peripheral cue was placed in the diametrically opposite position. In this report, peripheral cues were always located within 45° radial angle relative to the horizontal meridian and more than 5° in radial eccentricity from the central cue. Analysis of microsaccade rate and directionality in the 500 ms window surrounding cue onset revealed no differences depending on the location of the peripheral cue or depending on whether the peripheral cue remained illuminated or not. Accordingly, we pooled all trials together, subdividing data based only on the side of the cue relative to the side of FEF inactivation.

### Data Analysis

Offline, we screened all trials for microsaccades in a customized graphics user interface made in MatLab (Mathworks) that automatically detected microsaccade onset and offset using velocity (10°/s) and acceleration (600°/s^2^) criteria. We only accepted trials in which the monkey maintained fixation of the central cue for the full delayed period and removed any trial in which we identified any blinks or other aberrant changes in eye position or velocity (e.g., due to fatigue or inattention). We verified the onset and offset marks for each microsaccade and removed any microsaccades with amplitudes greater than 3° or severe curvatures in their trajectories (i.e., ratio of maximal to final displacement greater than 2). To differentiate microsaccades from drift, we also removed any microsaccades with onset accelerations lower than 1,000°/s^2^. We considered all microsaccades generated for each monkey actively fixating the central cue (i.e., fixation and delayed response periods) regardless of whether they correctly looked to the location of the peripheral cue. Similar results were observed if we constrained our analysis only to successfully performed trials. While our amplitude limit of 3° is very liberal, we wanted to ensure that any reduction of microsaccade occurrence during FEF inactivation (see Results) was not due to a coinciding increase in microsaccade amplitude above an arbitrary limit. Despite this liberal definition of microsaccade amplitude, and despite the specifics of our task and fixation window size, for each monkey, we found that the distribution of microsaccade amplitudes (e.g., median microsaccade amplitude of 0.51° for our example monkey in the FEF warm condition, see Fig 1A) was in good agreement with previous studies in monkeys and humans (reviewed in [62]). Perhaps most importantly, all of the results of FEF inactivation still held if we reduced our amplitude limit to 2°.

We investigated the contribution of the FEF to multiple aspects of microsaccade behavior in this manuscript. Microsaccade rate was defined as the number of microsaccades within a sliding ±50 ms rectangular window (in steps of 5 ms) divided by the number of all acceptable trials. Based on observations across monkeys, we used fixed time windows to quantify the microsaccade rate for the pre-cue period (i.e., 200 ms preceding cue onset), microsaccadic inhibition period (i.e., 60–140 ms after cue onset), and rate rebound period (i.e., 140–400 ms after cue onset; see Fig 3B for depiction of these periods). In order to investigate the timing of cue-induced microsaccades, we defined the microsaccade response time as the mean latency of the first microsaccade generated following cue onset during the rate rebound period. We defined the microsaccade amplitude as the angular vectorial displacement from microsaccade onset to offset. The microsaccade peak velocity was defined as the maximal vectorial velocity during its movement. To characterize changes in peak velocity, we constructed velocity-amplitude main sequence relationships and then extracted the peak velocity for 2° microsaccades from a fitted linear regression. We also investigated microsaccade directionality as the fraction of microsaccades toward the cue (i.e., sum of microsaccades toward the cue divided by the sum of microsaccades directed either toward or away from the cue); therefore, microsaccade directionality was independent of rate. Microsaccades directed within ±45° of the cue or diametrically opposite location of the cue were classified as toward or away from the cue, respectively. Finally, we also determined the specific timing of the microsaccade rate signature for each monkey. For this analysis, we first counted microsaccades across the full trial duration in ±50 ms bins and then calculated a threshold number of microsaccades that corresponded to 20% of the mean number in the pre-cue period. We determined the start of microsaccadic inhibition and rebound periods by incrementing bins backward and forward from 100 ms after cue onset in 1 ms steps, respectively, to find the next bin that exceeded the threshold number.

To determine the time course and statistics of microsaccade rate and directionality, we performed sliding window analyses in which we calculated a given measure within a ±50 ms window, and incrementally shifted this window every 5 ms for the full trial duration. The 95% confidence intervals of the mean microsaccade rate and peak velocity at 2° were calculated using 5,000 bootstrapped samples of randomly selected trials with replacement, while for directionality we used a binomial probability function. For statistical comparisons of specific time periods and/or conditions between bootstrapped distributions, we performed Welch's *t* tests (*p* < 0.05). For all other microsaccade measures, we determined statistical significance using Wilcoxon rank sum tests (*p* < 0.05).

## Supporting Information

S1 Data Data for Fig 1. (XLSX)

S2 Data Data for Fig 2. (XLSX)

S3 Data Data for Fig 3. (XLSX)

S4 Data Data for Fig 4. (XLSX)

S5 Data Data for Fig 5. (XLSX)

S6 Data Data for Fig 6. (XLSX)

S7 Data Data for S1 Fig. (XLSX)

S8 Data Data for S2 Fig. (XLSX)

S9 Data Data for S3 Fig. (XLSX)

S10 Data Data for S4 Fig. (XLSX)

S11 Data Data for S5 Fig. (XLSX)

S1 Fig FEF inactivation biased fixation position. (**A**) Unilateral FEF inactivation biased fixation position toward the intact side. Mean horizontal and vertical eye position in pre-cue period for FEF warm and FEF cool trials from our example monkey DZ with a unilateral (left) FEF inactivation after removing any outliers (>3 standard deviation). Lines indicate the mean +/- standard deviation for each condition. (**B**) This bias in horizontal eye position toward the intact side (+/- standard error) during FEF inactivation was largely stable before and after cue onset. (**C**) Consistent horizontal biases toward the intact side occurred for each monkey (GB, DZ, and OZ) and unilateral (X^r^ or X^l^) inactivation configuration, whereas bilateral FEF inactivation (X^bi^) consistently biased fixation positions to one affected side. All differences in position offset were statistically significant using a Wilcoxon rank sum test (*p* < 0.05). Data in Supporting Information (see S7 Data).
(TIF)

S2 Fig FEF inactivation prolonged the onset of the first microsaccade in the rebound period. (**A**) Number of the first rebound microsaccades across pre-, peri-, and post-cooling trials from our example monkey DZ. FEF inactivation increased the response time for microsaccades specifically occurring within the rebound period. Vertical lines indicate the mean response time for rebound microsaccades. (**B**) Microsaccadic response time increased across monkeys in three of five unilateral inactivation configurations, whereas bilateral FEF inactivation produced a quantitatively larger and more consistent increase in microsaccadic response time. Same format as S1C Fig. Data in Supporting Information (see S8 Data).
(TIF)

S3 Fig FEF inactivation did not influence drift velocity before cue onset. (**A**) Unilateral (left) inactivation had no effect on radial drift velocity within the 750 ms before cue onset in our example monkey DZ. For this analysis, we calculated the mean radial velocity from each trial after removing any intervals with microsaccades (10 ms before to 10 ms after) and artifacts (radial velocity >20°/s). (**B**) Across monkeys, FEF inactivation did not significantly influence radial drift velocity with absolute differences always less than 0.25°/s. Same format as S1C Fig. Note that our eye tracker was not well suited to study drift at a higher resolution; thus, it is possible that FEF inactivation caused effects on drift beyond the limits of our eye tracking technology. Data in Supporting Information (see S9 Data).
(TIF)

S4 Fig FEF inactivation had no effect on the post-microsaccadic increase in drift velocity. (**A**) Radial drift velocity somewhat increased following microsaccades in our example monkey DZ, but FEF inactivation did not alter this relationship. Pre- and post-microsaccade radial drift velocity were calculated from 60 to 10 ms before microsaccade onset and 10 to 60 ms after microsaccade offset, respectively, although we first removed any time points with artifacts (radial velocity >20°/s). (**B**) Across monkeys, we observed a similar post-microsaccadic increase of radial drift velocity for FEF warm trials. While FEF inactivation sometimes produced significant effects on the post-microsaccadic increases (indicated by asterisks above differences, Wilcoxon rank sum test, *p* < 0.025), such effects were either marginal (<0.25°/s) or not consistently observed across monkeys. Same format as S1C Fig. Data in Supporting Information (see S10 Data).
(TIF)

S5 Fig Reducing the top-down drive in an existing model of cue-induced microsaccade deployment captures our experimentally observed effects on microsaccade rate. (**A** and **B**) Time courses of mean microsaccade rate (+/- 95% confidence intervals) in response to cues for unilateral and bilateral inactivation simulations, respectively. Microsaccade rate is shown for the normal model (red) and with parameter changes to reflect reduced FEF drive (blue). Our unilateral inactivation model implemented only a simple reduction in the facilitation factor (i.e., top-down drive) that is specific for rebound microsaccades, which delayed and reduced their occurrence after cue onset, similar to our experimental results (see Fig 5A). The bilateral inactivation model additionally implemented a reduction in overall drive for all microsaccades and simulated both a decrease in pre-cue microsaccade rate and a further blunting of rate for rebound microsaccades comparable to the effects of bilateral FEF inactivation (see Fig 5B). Note that both models simulated identical results for cues in either visual hemifield, and we used the same procedures to determine the time-course and statistics for our modeling data, except that we implemented a ±25 ms window instead, which more precisely represented our observed post-cue microsaccade modulations in FEF warm trials. Data in Supporting Information (see S11 Data).
(TIF)

S1 Text Supplemental results. (DOCX)

## Abbreviations

FEF: frontal eye fields
iSC: intermediate layers of the superior colliculus
SC: superior colliculus
