# Supplementary figures and images for: A Causal Role for the Cortical Frontal Eye Fields in Microsaccade Deployment
Source: PLoS Biol. 2016 Aug 10;14(8):e1002531. doi: 10.1371/journal.pbio.1002531 (PMC4980061; doi:10.1371/journal.pbio.1002531)

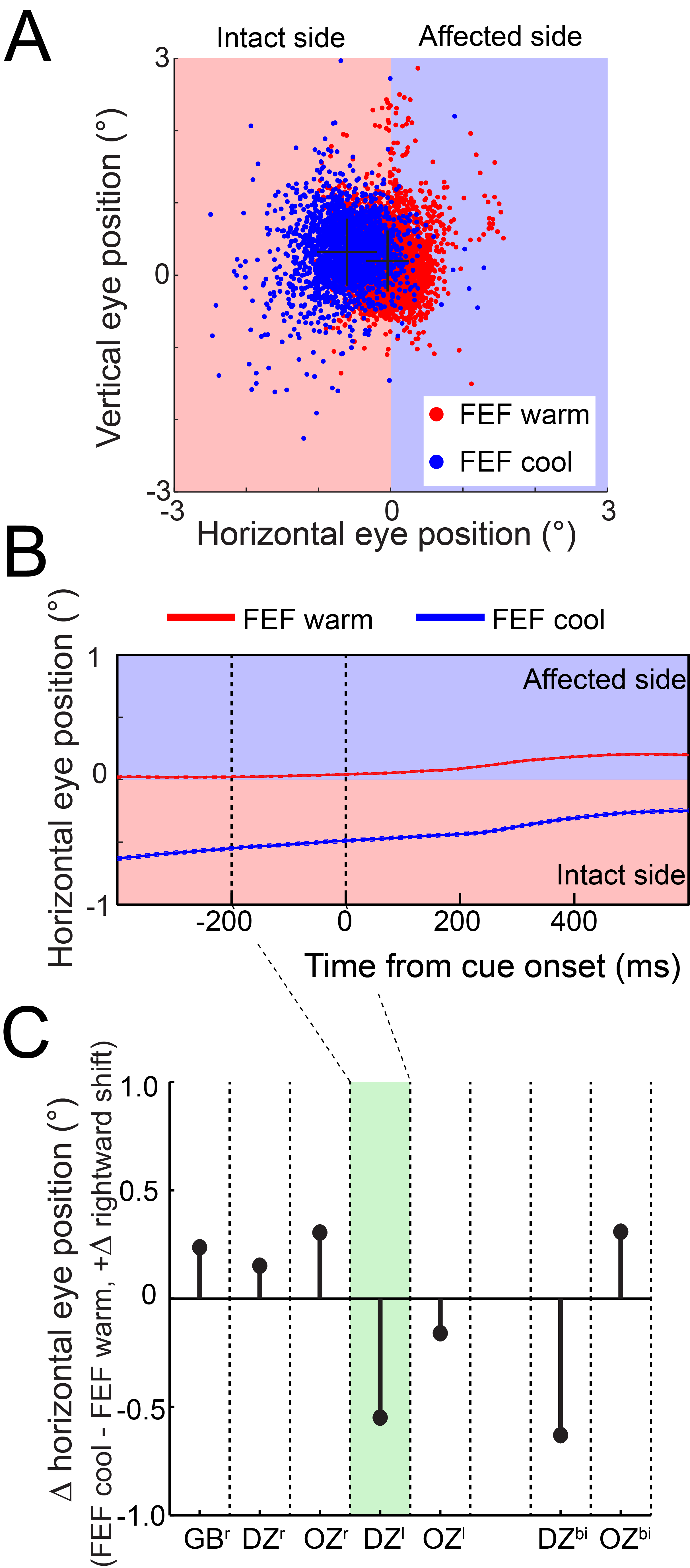

Supplement: S1 Fig — (A) Unilateral FEF inactivation biased fixation position toward the intact side. Mean horizontal and vertical eye position in pre-cue period for FEF warm and FEF cool trials from our example monkey DZ with a unilateral (left) FEF inactivation after removing any outliers (>3 standard deviation). Lines indicate the mean +/- standard deviation for each condition. (B) This bias in horizontal eye position toward the intact side (+/- standard error) during FEF inactivation was largely stable before and after cue onset. (C) Consistent horizontal biases toward the intact side occurred for each monkey (GB, DZ, and OZ) and unilateral (Xr or Xl) inactivation configuration, whereas bilateral FEF inactivation (Xbi) consistently biased fixation positions to one affected side. All differences in position offset were statistically significant using a Wilcoxon rank sum test (p < 0.05). Data in Supporting Information (see S7 Data). (TIF) [file pbio.1002531.s012.tif]

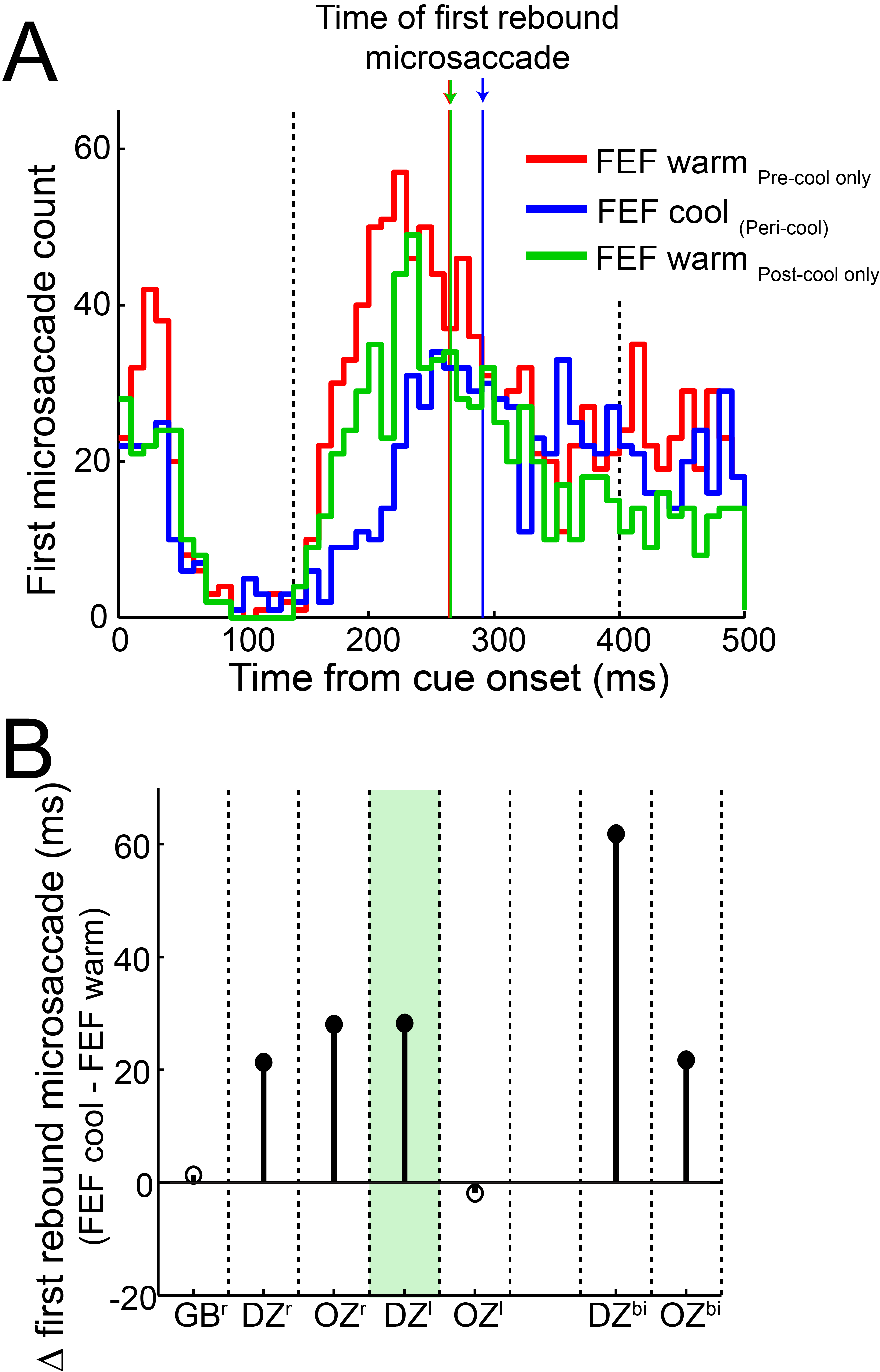

Supplement: S2 Fig — (A) Number of the first rebound microsaccades across pre-, peri-, and post-cooling trials from our example monkey DZ. FEF inactivation increased the response time for microsaccades specifically occurring within the rebound period. Vertical lines indicate the mean response time for rebound microsaccades. (B) Microsaccadic response time increased across monkeys in three of five unilateral inactivation configurations, whereas bilateral FEF inactivation produced a quantitatively larger and more consistent increase in microsaccadic response time. Same format as S1C Fig. Data in Supporting Information (see S8 Data). (TIF) [file pbio.1002531.s013.tif]

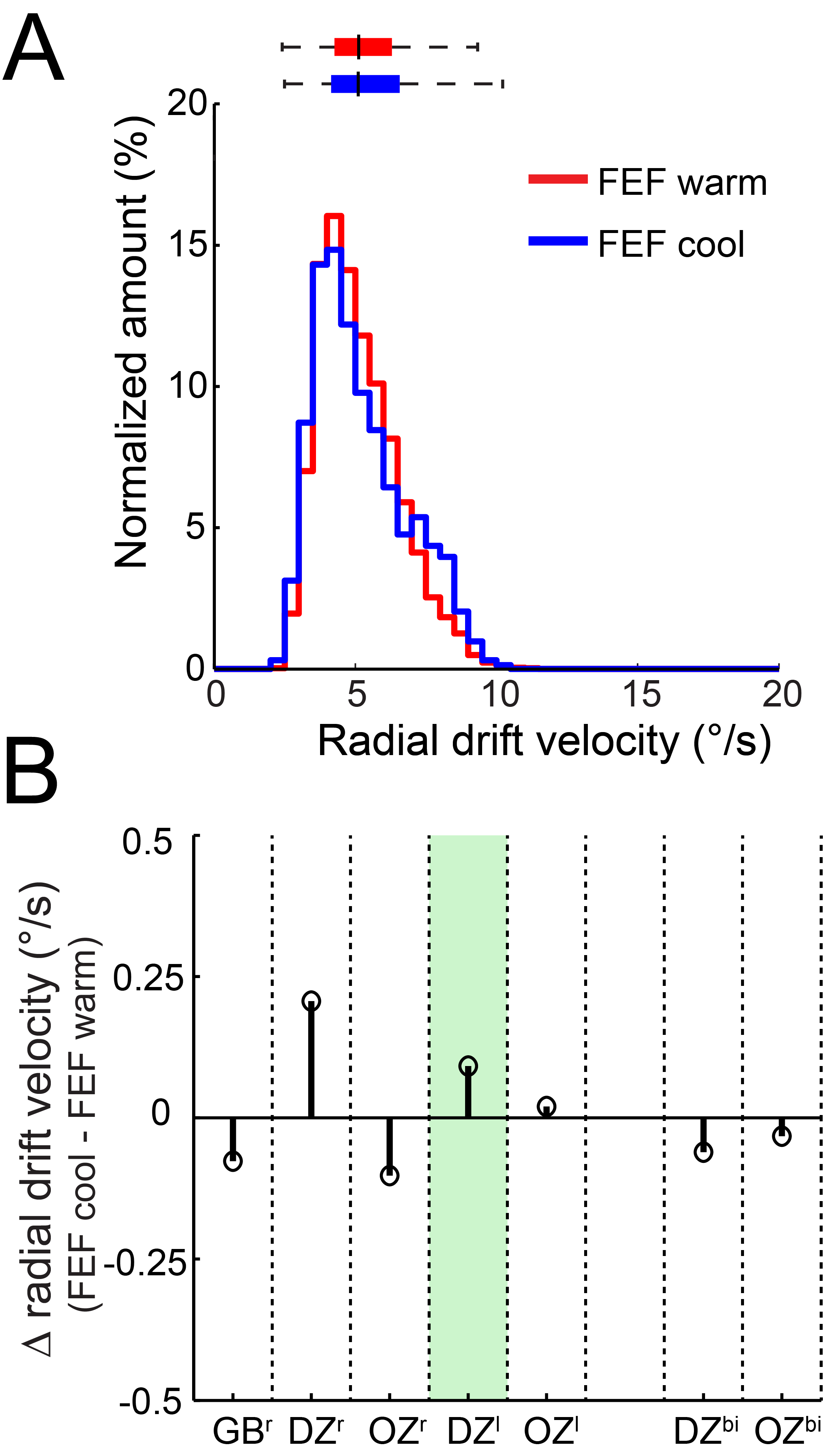

Supplement: S3 Fig — (A) Unilateral (left) inactivation had no effect on radial drift velocity within the 750 ms before cue onset in our example monkey DZ. For this analysis, we calculated the mean radial velocity from each trial after removing any intervals with microsaccades (10 ms before to 10 ms after) and artifacts (radial velocity >20°/s). (B) Across monkeys, FEF inactivation did not significantly influence radial drift velocity with absolute differences always less than 0.25°/s. Same format as S1C Fig. Note that our eye tracker was not well suited to study drift at a higher resolution; thus, it is possible that FEF inactivation caused effects on drift beyond the limits of our eye tracking technology. Data in Supporting Information (see S9 Data). (TIF) [file pbio.1002531.s014.tif]

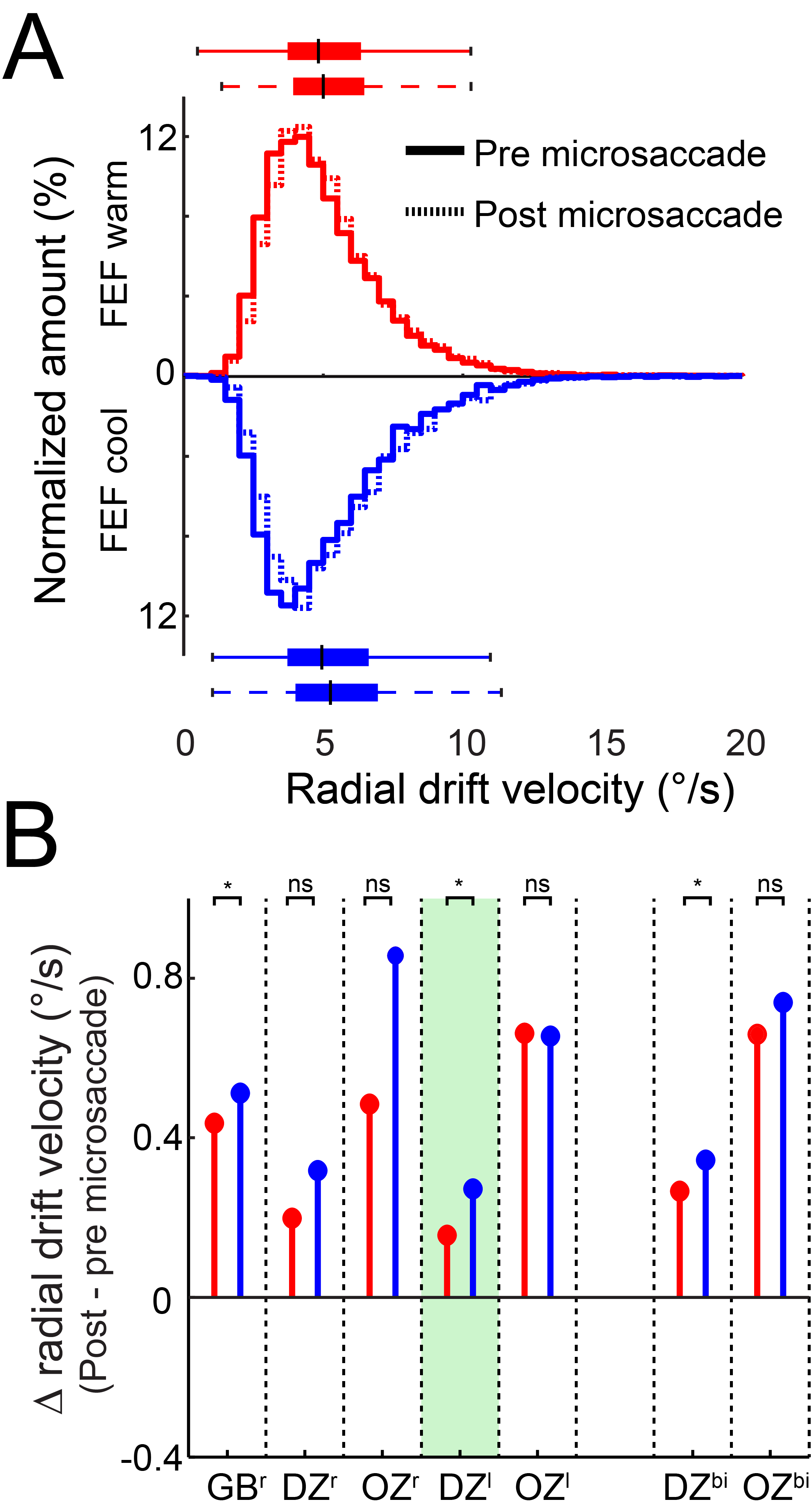

Supplement: S4 Fig — (A) Radial drift velocity somewhat increased following microsaccades in our example monkey DZ, but FEF inactivation did not alter this relationship. Pre- and post-microsaccade radial drift velocity were calculated from 60 to 10 ms before microsaccade onset and 10 to 60 ms after microsaccade offset, respectively, although we first removed any time points with artifacts (radial velocity >20°/s). (B) Across monkeys, we observed a similar post-microsaccadic increase of radial drift velocity for FEF warm trials. While FEF inactivation sometimes produced significant effects on the post-microsaccadic increases (indicated by asterisks above differences, Wilcoxon rank sum test, p < 0.025), such effects were either marginal (<0.25°/s) or not consistently observed across monkeys. Same format as S1C Fig. Data in Supporting Information (see S10 Data). (TIF) [file pbio.1002531.s015.tif]

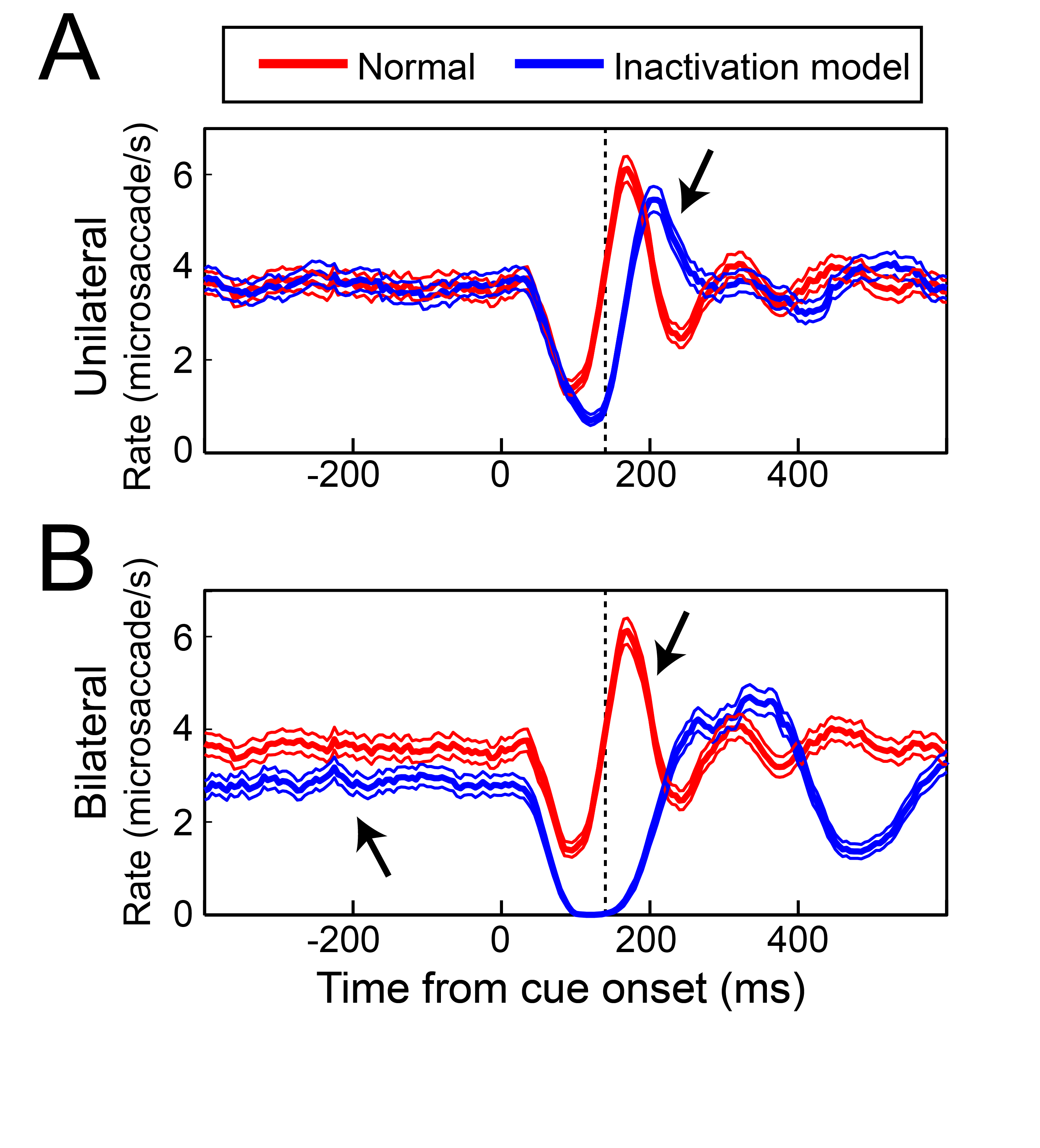

Supplement: S5 Fig — (A and B) Time courses of mean microsaccade rate (+/- 95% confidence intervals) in response to cues for unilateral and bilateral inactivation simulations, respectively. Microsaccade rate is shown for the normal model (red) and with parameter changes to reflect reduced FEF drive (blue). Our unilateral inactivation model implemented only a simple reduction in the facilitation factor (i.e., top-down drive) that is specific for rebound microsaccades, which delayed and reduced their occurrence after cue onset, similar to our experimental results (see Fig 5A). The bilateral inactivation model additionally implemented a reduction in overall drive for all microsaccades and simulated both a decrease in pre-cue microsaccade rate and a further blunting of rate for rebound microsaccades comparable to the effects of bilateral FEF inactivation (see Fig 5B). Note that both models simulated identical results for cues in either visual hemifield, and we used the same procedures to determine the time-course and statistics for our modeling data, except that we implemented a ±25 ms window instead, which more precisely represented our observed post-cue microsaccade modulations in FEF warm trials. Data in Supporting Information (see S11 Data). (TIF) [file pbio.1002531.s016.tif]
