# Supplementary material for: A Causal Role for the Cortical Frontal Eye Fields in Microsaccade Deployment
Source: PLoS Biol. 2016 Aug 10;14(8):e1002531. doi: 10.1371/journal.pbio.1002531 (PMC4980061; doi:10.1371/journal.pbio.1002531)
Supplement: S1 Text — (DOCX) [file pbio.1002531.s017.docx]

**S1 Text. Supplemental Results**

***Simulating the effects of reducing top-down drive in a model of microsaccade deployment***

To help interpret our results, we wondered whether an existing model of cue-induced microsaccade deployment by Hafed and Ignashchenkova [1] could be modified to produce microsaccade rate results similar to what we observed during FEF inactivation. According to the model [1], cue onsets influence microsaccades in a manner similar to how sudden visual transients influence large saccades [2]: resetting ongoing saccadic activity, which subsequently recovers to its normal behavior. Implied in this model is the idea that microsaccades immediately after cue onset are generated by a reflexive bottom-up mechanism, whereas the latter microsaccades in the rebound period are produced by top-down recovery from this cue-induced disruption. Moreover, these rebound microsaccades have a faster rate of rise to threshold, due to a ‘facilitation factor’, compared to other microsaccades. Given this framework, we tested whether reducing the top-down drive in the model, and hence slowing the rate of rise of rebound microsaccades, could reproduce the experimentally-observed effects of FEF inactivation on rebound microsaccades.

To simulate a reduced top-down drive, we created a unilateral inactivation model in which we scaled down the model’s facilitation factor for rebound microsaccades (r_B_ in the normal model by a factor of 0.65). We predicted that this single parameter change would be sufficient to generate microsaccade rate modulations similar to our unilateral FEF inactivation experiments. This prediction is supported by **S5A Fig**, in which we simulated the original model (red) and the unilateral inactivation model with reduced top-down drive (blue) for 4,000 trials each. As can be seen, the modified model captured the delay and reduction of rebound microsaccades that we observed experimentally during FEF inactivation (compare to **Fig 3B** and **Fig 5A**). Notably, this model does not introduce any directional biases, and it thus produced similar effects regardless of where the cue appeared. Therefore, a reduced top-down drive within the model's framework is consistent with the effects of FEF inactivation on rebound microsaccades.

Since we altered a single parameter that specifically affects rebound microsaccades, our unilateral inactivation model did not perturb microsaccade rate before cue onset, consistent with our experimental results. However, bilateral FEF inactivation decreased pre-cue microsaccade rate, thus we hypothesized that a general decrease in the overall FEF drive, presumably due to the larger cortical inactivation, could account for these discrepancies. We tested this in our bilateral inactivation model with the same modification that reduced top-down drive as in the unilateral inactivation model, but we also scaled down the slope in the rise-to-threshold process for all microsaccades independent of when they occurred (*i.e.,* this slope reduction to 0.65 of the normal model influenced both pre-cue and rebound microsaccades). Critically, the bilateral inactivation model captured both the decrement in pre-cue microsaccade rate, and a further blunting of rebound microsaccade rate (**S5B Fig**), similar to our experimental manipulations (compare to **Fig 5B**). Accordingly, our modified models with reduced top-down drive are conceptually compatible with the decreased rate of microsaccades during FEF inactivation, particularly after cue onset.

**References**

1. Hafed ZM, Ignashchenkova A. On the dissociation between microsaccade rate and direction after peripheral cues: microsaccadic inhibition revisited. J Neurosci. 2013;33: 16220–35. doi:10.1523/JNEUROSCI.2240-13.2013

2. Reingold EM, Stampe DM. Saccadic inhibition in voluntary and reflexive saccades. J Cogn Neurosci. 2002;14: 371–388. doi:10.1162/089892902317361903
